# Tetra­kis(μ-4-azido­benzoato-κ^2^ *O*:*O*′)bis­[(*N*,*N*-dimethyl­formamide-κ*O*)copper(II)]

**DOI:** 10.1107/S1600536811050999

**Published:** 2011-12-14

**Authors:** Aidong Wang

**Affiliations:** a Department of Chemistry, Huangshan University, Huangshan 245041, People’s Republic of China

## Abstract

The binuclear title compound, [Cu_2_(C_7_H_4_N_3_O_2_)_4_(C_3_H_7_NO)_2_], is a discrete metal–organic compound having a paddle-wheel-type structure. The Cu⋯Cu distance is 2.6366 (5) Å and an inversion center is located at the mid-point of this bond. The Cu^II^ cation is coordinated by four carboxyl­ate O atoms from four 4-azido­benzoate ligands, and one O atom from a dimethyl­formamide mol­ecule, forming an overall distorted octahedral geometry when the Cu⋯Cu bond is also considered.

## Related literature

For similar complexes displaying a paddle-wheel structure, see: Del Sesto *et al.* (2000 ▶); Li *et al.* (2011 ▶). For the synthesis of 4-azido­benzoic acid, see: Sato *et al.* (2010 ▶).
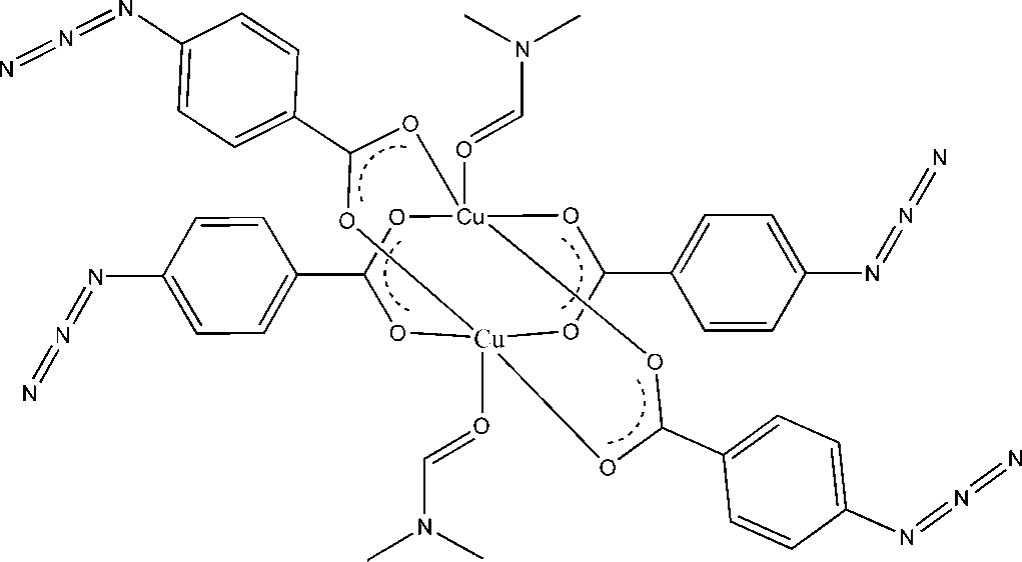


## Experimental

### 

#### Crystal data

[Cu_2_(C_7_H_4_N_3_O_2_)_4_(C_3_H_7_NO)_2_]
*M* *_r_* = 921.80
Monoclinic, 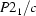

*a* = 11.9209 (8) Å
*b* = 17.9387 (10) Å
*c* = 9.3680 (5) Å
β = 91.277 (5)°
*V* = 2002.8 (2) Å^3^
*Z* = 2
Mo *K*α radiation
μ = 1.14 mm^−1^
*T* = 293 K
0.2 × 0.2 × 0.2 mm

#### Data collection

Rigaku Mercury70 diffractometer
Absorption correction: multi-scan (*CrystalClear*; Rigaku, 2002 ▶) *T*_min_ = 0.805, *T*_max_ = 0.805
12312 measured reflections
3496 independent reflections
3141 reflections with *I* > 2σ(*I*)
*R*_int_ = 0.031

#### Refinement

*R*[*F* ^2^ > 2σ(*F* ^2^)] = 0.033
*wR*(*F* ^2^) = 0.091
*S* = 1.02
3496 reflections
271 parameters
H-atom parameters constrained
Δρ_max_ = 0.33 e Å^−3^
Δρ_min_ = −0.31 e Å^−3^

### 

Data collection: *CrystalClear* (Rigaku, 2002 ▶); cell refinement: *CrystalClear*; data reduction: *CrystalClear*; program(s) used to solve structure: *SHELXS97* (Sheldrick, 2008 ▶); program(s) used to refine structure: *SHELXL97* (Sheldrick, 2008 ▶); molecular graphics: *SHELXTL* (Sheldrick, 2008 ▶); software used to prepare material for publication: *SHELXL97*.

## Supplementary Material

Crystal structure: contains datablock(s) I, global. DOI: 10.1107/S1600536811050999/bh2402sup1.cif

Structure factors: contains datablock(s) I. DOI: 10.1107/S1600536811050999/bh2402Isup2.hkl

Additional supplementary materials:  crystallographic information; 3D view; checkCIF report

## Figures and Tables

**Fig. 1.**
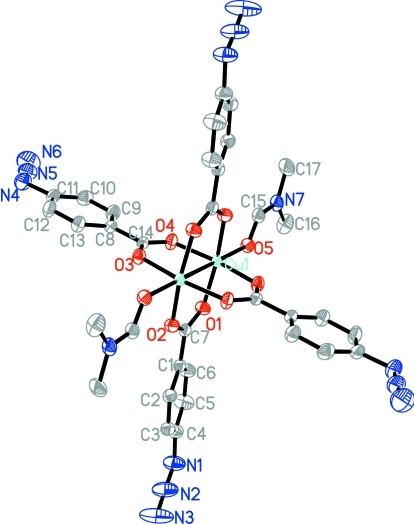
A molecular drawing of the title complex, showing 30% probability displacement ellipsoids. All H atoms were omitted for clarity. Unlabeled atoms are generated by symmetry -x, 1-y, 1-z.

## supplementary crystallographic information

### Comment

The title compound, tetrakis(4-azidobenzoato)bis[(*N*,*N*-dimethylformamide)copper(II)], crystallizes in the monoclinic form with centrosymmetric space group *P*2_1_/c. The asymmetric unit contains one copper atom, two 4-azidobenzoate ligands, and one DMF molecule. The Cu atom has a coordination number of six and is coordinated by four carboxylate O atoms from four 4-azidobenzoate ligands and one O atom from DMF molecule. The Cu—Cu bond length is 2.6366 (5) Å. The main structural feature of the title compound is the presence of the well known paddle-wheel unit (Del Sesto *et al.*, 2000; Li *et al.*, 2011) constructed by the asymmetric unit *via* the inversion symmetry. The azido groups of the ligands are not coordinated and the axial positions of the octahedral coordination polyhedra are occupied by two DMF molecules, to generate the 0D compound.

### Experimental

4-Azidobenzoic acid was prepared from 4-aminoisophthalic acid by diazotization followed by azidation with sodium azide (Sato *et al.*, 2010). A mixture of Cu(NO_3_)_2 _3 H_2_O (0.130 g, 0.5 mmol) 4-azidobenzoic acid (0.085 g,0.5 mmol) and DMF (5 ml) was sealed in a 20 ml stainless steel reactor with Teflon liner and heated at 393 K for 4 days. Blue crystals of the title complex were obtained.

### Refinement

H atoms bonded to C atoms were positioned geometrically. C—H bonds lengths were fixed at 0.93 Å for aromatic CH groups and 0.96 Å for methyl groups, and H atoms were allowed to ride on their parent atoms. Isotropic displacement parameters were calculated as *U*_iso_(H)=1.2*U*_eq_(carrier C) for aromatic CH and *U*_iso_(H)=1.5*U*_eq_(carrier C) for methyl groups.

### Crystal data

**Table:**

[Cu_2_(C_7_H_4_N_3_O_2_)_4_(C_3_H_7_NO)_2_]	*F*(000) = 940
*M**_r_* = 921.80	*D*_x_ = 1.529 Mg m^−^^3^
Monoclinic, *P*2_1_/*c*	Mo *K*α radiation, λ = 0.71073 Å
Hall symbol: -P 2ybc	Cell parameters from 4783 reflections
*a* = 11.9209 (8) Å	θ = 3.1–25°
*b* = 17.9387 (10) Å	µ = 1.14 mm^−^^1^
*c* = 9.3680 (5) Å	*T* = 293 K
β = 91.277 (5)°	Block, blue
*V* = 2002.8 (2) Å^3^	0.2 × 0.2 × 0.2 mm
*Z* = 2	

### Data collection

**Table:**

Rigaku Mercury70 diffractometer	3496 independent reflections
Radiation source: fine-focus sealed tube	3141 reflections with *I* > 2σ(*I*)
graphite	*R*_int_ = 0.031
Detector resolution: 14.6306 pixels mm^-1^	θ_max_ = 25.0°, θ_min_ = 2.5°
ω scans	*h* = −14→14
Absorption correction: multi-scan (*CrystalClear*; Rigaku, 2002)	*k* = −19→21
*T*_min_ = 0.805, *T*_max_ = 0.805	*l* = −11→11
12312 measured reflections	

### Refinement

**Table:**

Refinement on *F*^2^	Primary atom site location: structure-invariant direct methods
Least-squares matrix: full	Secondary atom site location: difference Fourier map
*R*[*F*^2^ > 2σ(*F*^2^)] = 0.033	Hydrogen site location: inferred from neighbouring sites
*wR*(*F*^2^) = 0.091	H-atom parameters constrained
*S* = 1.02	*w* = 1/[σ^2^(*F*_o_^2^) + (0.0482*P*)^2^ + 0.7327*P*] where *P* = (*F*_o_^2^ + 2*F*_c_^2^)/3
3496 reflections	(Δ/σ)_max_ = 0.001
271 parameters	Δρ_max_ = 0.33 e Å^−^^3^
0 restraints	Δρ_min_ = −0.31 e Å^−^^3^
0 constraints	

### Fractional atomic coordinates and isotropic or equivalent isotropic displacement parameters (Å^2^)

**Table:**

	*x*	*y*	*z*	*U*_iso_*/*U*_eq_	
Cu1	0.05068 (2)	0.451211 (14)	0.58459 (3)	0.03461 (12)	
O1	0.16267 (14)	0.44589 (9)	0.43570 (18)	0.0472 (4)	
O5	0.12483 (14)	0.36844 (9)	0.72800 (17)	0.0470 (4)	
O3	0.04281 (13)	0.62087 (9)	0.52445 (18)	0.0461 (4)	
O2	0.07599 (14)	0.52740 (10)	0.28944 (17)	0.0467 (4)	
O4	0.12931 (15)	0.53807 (9)	0.66805 (19)	0.0497 (4)	
N7	0.1214 (2)	0.31119 (12)	0.9451 (2)	0.0567 (6)	
N1	0.4690 (2)	0.4056 (2)	−0.1016 (3)	0.0854 (9)	
N2	0.4467 (3)	0.4246 (2)	−0.2269 (4)	0.1045 (11)	
N6	0.4782 (4)	0.8193 (3)	1.0817 (4)	0.1397 (18)	
N3	0.4367 (4)	0.4395 (3)	−0.3436 (4)	0.155 (2)	
N4	0.3389 (3)	0.84292 (18)	0.9011 (3)	0.0855 (9)	
N5	0.4106 (3)	0.8267 (2)	0.9946 (3)	0.0987 (12)	
C4	0.3881 (2)	0.42884 (18)	−0.0005 (3)	0.0584 (7)	
C7	0.15287 (19)	0.48163 (13)	0.3208 (2)	0.0398 (5)	
C15	0.0863 (2)	0.35912 (14)	0.8475 (3)	0.0484 (6)	
H15A	0.0259	0.3892	0.8714	0.058*	
C1	0.2378 (2)	0.46654 (13)	0.2094 (3)	0.0414 (5)	
C8	0.17273 (19)	0.66528 (13)	0.6976 (2)	0.0409 (5)	
C2	0.2172 (2)	0.48554 (14)	0.0677 (2)	0.0460 (6)	
H2A	0.1524	0.5118	0.0430	0.055*	
C6	0.3359 (2)	0.42912 (18)	0.2440 (3)	0.0595 (7)	
H6A	0.3512	0.4159	0.3384	0.071*	
C13	0.1512 (2)	0.73877 (15)	0.6624 (3)	0.0539 (6)	
H13A	0.0985	0.7498	0.5907	0.065*	
C3	0.2911 (2)	0.46609 (15)	−0.0371 (3)	0.0512 (6)	
H3A	0.2753	0.4781	−0.1320	0.061*	
C9	0.2511 (2)	0.65078 (16)	0.8053 (3)	0.0556 (7)	
H9A	0.2661	0.6016	0.8308	0.067*	
C14	0.11051 (19)	0.60338 (13)	0.6237 (2)	0.0397 (5)	
C10	0.3073 (2)	0.70738 (18)	0.8752 (3)	0.0603 (7)	
H10A	0.3596	0.6966	0.9475	0.072*	
C12	0.2067 (3)	0.79604 (16)	0.7321 (3)	0.0635 (8)	
H12B	0.1908	0.8453	0.7081	0.076*	
C11	0.2856 (2)	0.78027 (17)	0.8373 (3)	0.0576 (7)	
C17	0.0706 (3)	0.3082 (2)	1.0850 (3)	0.0822 (11)	
H17A	0.0097	0.3432	1.0881	0.123*	
H17B	0.1258	0.3207	1.1572	0.123*	
H17C	0.0428	0.2588	1.1018	0.123*	
C16	0.2150 (3)	0.26297 (19)	0.9201 (4)	0.0807 (10)	
H16A	0.2407	0.2704	0.8248	0.121*	
H16B	0.1923	0.2120	0.9315	0.121*	
H16C	0.2746	0.2742	0.9873	0.121*	
C5	0.4114 (2)	0.4111 (2)	0.1403 (3)	0.0717 (9)	
H5A	0.4781	0.3871	0.1652	0.086*	

### Atomic displacement parameters (Å^2^)

**Table:**

	*U*^11^	*U*^22^	*U*^33^	*U*^12^	*U*^13^	*U*^23^
Cu1	0.04444 (19)	0.02783 (19)	0.03131 (18)	0.00240 (10)	−0.00460 (12)	0.00345 (10)
O1	0.0503 (10)	0.0503 (11)	0.0409 (9)	0.0099 (7)	0.0020 (7)	0.0104 (8)
O5	0.0606 (10)	0.0393 (10)	0.0408 (9)	0.0062 (8)	−0.0076 (7)	0.0085 (8)
O3	0.0538 (10)	0.0364 (10)	0.0474 (9)	−0.0021 (7)	−0.0126 (8)	0.0021 (8)
O2	0.0541 (10)	0.0447 (10)	0.0416 (9)	0.0133 (8)	0.0052 (7)	0.0058 (8)
O4	0.0648 (11)	0.0328 (10)	0.0507 (10)	−0.0040 (8)	−0.0167 (8)	0.0013 (8)
N7	0.0779 (15)	0.0430 (13)	0.0485 (12)	−0.0044 (11)	−0.0166 (11)	0.0141 (10)
N1	0.0804 (18)	0.112 (3)	0.0643 (18)	0.0276 (17)	0.0221 (14)	−0.0009 (17)
N2	0.088 (2)	0.157 (3)	0.069 (2)	0.035 (2)	0.0240 (17)	−0.016 (2)
N6	0.135 (3)	0.192 (5)	0.091 (3)	−0.087 (3)	−0.021 (2)	−0.024 (3)
N3	0.131 (3)	0.272 (7)	0.061 (2)	0.059 (3)	0.026 (2)	−0.009 (3)
N4	0.094 (2)	0.088 (2)	0.0738 (18)	−0.0434 (17)	0.0006 (16)	−0.0252 (16)
N5	0.105 (2)	0.124 (3)	0.0677 (19)	−0.068 (2)	0.0097 (18)	−0.0299 (19)
C4	0.0564 (16)	0.0638 (19)	0.0553 (16)	0.0072 (14)	0.0110 (13)	−0.0019 (14)
C7	0.0454 (13)	0.0319 (13)	0.0421 (13)	0.0001 (10)	−0.0022 (10)	−0.0011 (10)
C15	0.0594 (15)	0.0403 (15)	0.0450 (14)	−0.0014 (11)	−0.0108 (11)	0.0081 (11)
C1	0.0471 (13)	0.0353 (13)	0.0418 (13)	0.0009 (10)	0.0004 (10)	0.0003 (10)
C8	0.0455 (12)	0.0370 (13)	0.0403 (12)	−0.0018 (10)	0.0023 (10)	−0.0048 (10)
C2	0.0527 (14)	0.0416 (14)	0.0437 (13)	0.0080 (11)	−0.0016 (10)	0.0015 (11)
C6	0.0550 (16)	0.076 (2)	0.0476 (15)	0.0150 (14)	−0.0009 (12)	0.0108 (14)
C13	0.0583 (15)	0.0437 (16)	0.0593 (16)	0.0019 (12)	−0.0070 (12)	−0.0032 (13)
C3	0.0627 (16)	0.0511 (16)	0.0398 (14)	−0.0009 (13)	−0.0006 (11)	−0.0011 (12)
C9	0.0623 (16)	0.0483 (16)	0.0557 (16)	−0.0008 (13)	−0.0105 (12)	−0.0027 (13)
C14	0.0473 (13)	0.0348 (13)	0.0370 (12)	−0.0003 (10)	0.0025 (10)	−0.0008 (10)
C10	0.0559 (16)	0.073 (2)	0.0517 (16)	−0.0075 (14)	−0.0087 (12)	−0.0109 (15)
C12	0.0780 (19)	0.0398 (16)	0.0725 (19)	−0.0082 (14)	−0.0040 (15)	−0.0064 (14)
C11	0.0612 (16)	0.0577 (18)	0.0543 (16)	−0.0193 (14)	0.0113 (13)	−0.0180 (14)
C17	0.124 (3)	0.076 (2)	0.0466 (17)	−0.021 (2)	−0.0081 (17)	0.0223 (15)
C16	0.098 (2)	0.059 (2)	0.084 (2)	0.0146 (18)	−0.0344 (19)	0.0090 (18)
C5	0.0555 (17)	0.097 (3)	0.0628 (18)	0.0284 (16)	0.0050 (14)	0.0108 (18)

### Geometric parameters (Å, °)

**Table:**

Cu1—O1	1.9545 (17)	C1—C2	1.387 (3)
Cu1—O4	1.9711 (17)	C1—C6	1.382 (4)
Cu1—O2^i^	1.9747 (16)	C8—C13	1.382 (4)
Cu1—O3^i^	1.9765 (16)	C8—C9	1.384 (3)
Cu1—O5	2.1767 (16)	C8—C14	1.496 (3)
Cu1—Cu1^i^	2.6363 (5)	C2—C3	1.379 (3)
O1—C7	1.256 (3)	C2—H2A	0.9300
O5—C15	1.231 (3)	C6—C5	1.377 (4)
O3—C14	1.258 (3)	C6—H6A	0.9300
O3—Cu1^i^	1.9765 (16)	C13—C12	1.379 (4)
O2—C7	1.260 (3)	C13—H13A	0.9300
O2—Cu1^i^	1.9747 (16)	C3—H3A	0.9300
O4—C14	1.261 (3)	C9—C10	1.375 (4)
N7—C15	1.316 (3)	C9—H9A	0.9300
N7—C16	1.436 (4)	C10—C11	1.378 (4)
N7—C17	1.456 (4)	C10—H10A	0.9300
N1—N2	1.245 (4)	C12—C11	1.376 (4)
N1—C4	1.429 (3)	C12—H12B	0.9300
N2—N3	1.130 (5)	C17—H17A	0.9600
N6—N5	1.142 (5)	C17—H17B	0.9600
N4—N5	1.245 (5)	C17—H17C	0.9600
N4—C11	1.417 (4)	C16—H16A	0.9600
C4—C3	1.373 (4)	C16—H16B	0.9600
C4—C5	1.379 (4)	C16—H16C	0.9600
C7—C1	1.495 (3)	C5—H5A	0.9300
C15—H15A	0.9300		
			
O1—Cu1—O4	89.73 (8)	C3—C2—C1	121.1 (2)
O1—Cu1—O2^i^	168.40 (7)	C3—C2—H2A	119.4
O4—Cu1—O2^i^	88.43 (8)	C1—C2—H2A	119.4
O1—Cu1—O3^i^	89.17 (7)	C5—C6—C1	120.8 (3)
O4—Cu1—O3^i^	168.48 (7)	C5—C6—H6A	119.6
O2^i^—Cu1—O3^i^	90.35 (7)	C1—C6—H6A	119.6
O1—Cu1—O5	97.62 (6)	C12—C13—C8	120.9 (3)
O4—Cu1—O5	96.40 (7)	C12—C13—H13A	119.6
O2^i^—Cu1—O5	93.97 (7)	C8—C13—H13A	119.6
O3^i^—Cu1—O5	95.12 (7)	C4—C3—C2	119.6 (2)
O1—Cu1—Cu1^i^	85.10 (5)	C4—C3—H3A	120.2
O4—Cu1—Cu1^i^	85.48 (5)	C2—C3—H3A	120.2
O2^i^—Cu1—Cu1^i^	83.34 (5)	C10—C9—C8	121.5 (3)
O3^i^—Cu1—Cu1^i^	83.00 (5)	C10—C9—H9A	119.2
O5—Cu1—Cu1^i^	176.69 (5)	C8—C9—H9A	119.2
C7—O1—Cu1	122.33 (15)	O3—C14—O4	125.6 (2)
C15—O5—Cu1	119.94 (16)	O3—C14—C8	117.4 (2)
C14—O3—Cu1^i^	124.34 (15)	O4—C14—C8	117.0 (2)
C7—O2—Cu1^i^	123.31 (15)	C9—C10—C11	119.4 (3)
C14—O4—Cu1	121.62 (15)	C9—C10—H10A	120.3
C15—N7—C16	121.1 (3)	C11—C10—H10A	120.3
C15—N7—C17	121.1 (3)	C11—C12—C13	120.0 (3)
C16—N7—C17	117.7 (3)	C11—C12—H12B	120.0
N2—N1—C4	114.4 (3)	C13—C12—H12B	120.0
N3—N2—N1	173.3 (4)	C12—C11—C10	120.1 (3)
N5—N4—C11	114.0 (3)	C12—C11—N4	115.6 (3)
N6—N5—N4	173.3 (4)	C10—C11—N4	124.3 (3)
C3—C4—C5	120.1 (2)	N7—C17—H17A	109.5
C3—C4—N1	123.6 (3)	N7—C17—H17B	109.5
C5—C4—N1	116.2 (3)	H17A—C17—H17B	109.5
O1—C7—O2	125.8 (2)	N7—C17—H17C	109.5
O1—C7—C1	117.0 (2)	H17A—C17—H17C	109.5
O2—C7—C1	117.1 (2)	H17B—C17—H17C	109.5
O5—C15—N7	126.9 (3)	N7—C16—H16A	109.5
O5—C15—H15A	116.5	N7—C16—H16B	109.5
N7—C15—H15A	116.5	H16A—C16—H16B	109.5
C2—C1—C6	118.4 (2)	N7—C16—H16C	109.5
C2—C1—C7	121.0 (2)	H16A—C16—H16C	109.5
C6—C1—C7	120.5 (2)	H16B—C16—H16C	109.5
C13—C8—C9	118.2 (2)	C4—C5—C6	120.0 (3)
C13—C8—C14	120.7 (2)	C4—C5—H5A	120.0
C9—C8—C14	121.1 (2)	C6—C5—H5A	120.0

Symmetry codes: (i) −*x*, −*y*+1, −*z*+1.
